# Humoral and cellular immunogenicity of homologous and heterologous booster vaccination in Ad26.COV2.S-primed individuals: Comparison by breakthrough infection

**DOI:** 10.3389/fimmu.2023.1131229

**Published:** 2023-03-07

**Authors:** Hakjun Hyun, A-Yeung Jang, Heedo Park, Jung Yeon Heo, Yu Bin Seo, Eliel Nham, Jin Gu Yoon, Hye Seong, Ji Yun Noh, Hee Jin Cheong, Woo Joo Kim, Soo-Young Yoon, Jong Hyeon Seok, Jineui Kim, Man-Seong Park, Joon Young Song

**Affiliations:** ^1^Division of Infectious Diseases, Department of Internal Medicine, Korea University College of Medicine, Seoul, Republic of Korea; ^2^Asia Pacific Influenza Institute, Korea University College of Medicine, Seoul, Republic of Korea; ^3^Department of Research and Development, Vaccine Innovation Center, Korea University College of Medicine, Seoul, Republic of Korea; ^4^Department of Microbiology, Institute for Viral Diseases, Biosafety center, College of Medicine, Korea University, Seoul, Republic of Korea; ^5^Department of Infectious Diseases, Ajou University School of Medicine, Suwon, Republic of Korea; ^6^Division of Infectious Disease, Department of Internal Medicine, Kangnam Sacred Heart Hospital, Hallym University College of Medicine, Seoul, Republic of Korea; ^7^Department of Laboratory Medicine, Korea University College of Medicine, Seoul, Republic of Korea

**Keywords:** SARS-CoV-2, COVID-19, vaccines, humoral immunity, cellular immunity, booster, breakthrough infection

## Abstract

**Background:**

Whether or not a single-dose Ad26.COV2.S prime and boost vaccination induces sufficient immunity is unclear. Concerns about the increased risk of breakthrough infections in the Ad26.COV2.S-primed population have also been raised.

**Methods:**

A prospective cohort study was conducted. Participants included healthy adults who were Ad26.COV2.S primed and scheduled to receive a booster vaccination with BNT162b2, mRNA-1273, or Ad26.COV2.S. The IgG anti-receptor binding domain (RBD) antibody titers, neutralizing antibody (NAb) titers (against wild type [WT] and Omicron [BA.1 and BA.5]), and Spike-specific interferon-γ responses of the participants were estimated at baseline, 3–4 weeks, 3 months, and 6 months after booster vaccination.

**Results:**

A total of 89 participants were recruited (26 boosted with BNT162b2, 57 with mRNA-1273, and 7 with Ad26.COV2.S). The IgG anti-RBD antibody titers of all participants were significantly higher at 6 months post-vaccination than at baseline. The NAb titers against WT at 3 months post-vaccination were 359, 258, and 166 in the participants from the BNT162b2-, mRNA-1273-, and Ad26.COV2.S-boosted groups, respectively. Compared with those against WT, the NAb titers against BA.1/BA.5 were lower by 23.9/10.9-, 16.6/7.4-, and 13.8/7.2-fold in the participants from the BNT162b2-, mRNA-1273-, and Ad26.COV2.S-boosted groups, respectively, at 3 months post-vaccination. Notably, the NAb titers against BA.1 were not boosted after Ad26.COV2.S vaccination. Breakthrough infections occurred in 53.8%, 62.5%, and 42.9% of the participants from the BNT162b2-, mRNA-1273-, and Ad26.COV2.S-boosted groups, respectively. No significant difference in humoral and cellular immunity was found between individuals with and without SARS-CoV-2 breakthrough infections.

**Conclusion:**

Booster vaccination elicited acceptable humoral and cellular immune responses in Ad26.COV2.S-primed individuals. However, the neutralizing activities against Omicron subvariants were negligible, and breakthrough infection rates were remarkably high at 3 months post-booster vaccination, irrespective of the vaccine type. A booster dose of a vaccine containing the Omicron variant antigen would be required.

## 1 Introduction

The coronavirus disease 2019 (COVID-19) pandemic has progressed since 2020. The humoral and cellular immunity elicited by vaccines is important to prevent disease transmission and progression (1–3). The spike (S) protein of severe acute respiratory syndrome coronavirus-2 (SARS-CoV-2) is the target of most commercial vaccines because of its crucial role in disease transmission (4). Mutations in the S protein, along with immune waning in the vaccinated population, have resulted in the immune evasion of the virus and breakthrough infections in vaccinated populations (5). A single dose of Ad26.COV2.S (Johnson & Johnson–Janssen, adenovirus-vectored vaccine) is immunogenic and has shown acceptable efficacy in clinical trials (6). However, Ad26.COV2.S vaccination is currently considered only when messenger RNA (mRNA) vaccines (BNT162b2 [Pfizer–BioNTech] and mRNA-1273 [Moderna–NIAID]) or protein-conjugated vaccines (Nuvaxovid [Novavax]) are unavailable. In addition, mRNA vaccines are preferred over Ad26.COV2.S for booster shots (7). Therefore, heterologous booster vaccination is generally employed for the Ad26.COV2.S-primed population.

Recent studies on COVID-19 vaccine effectiveness have reported that homologous mRNA booster vaccination is the most effective strategy. However, heterologous booster vaccination, i.e., adenovirus-vectored vaccine priming followed by mRNA vaccine booster administration, also shows acceptable effectiveness even against the Omicron (B.1.1.529 [BA.1]) variant (8).

As of November 2022, approximately 630 million confirmed cases of COVID-19 were recorded worldwide. Many vaccines based on diverse platforms have been developed to mitigate the COVID-19 pandemic. As of September 2022, the cumulative number of administered COVID-19 vaccine doses was approximately 12.6 billion (9). Thus, the “hybrid immunity” or the immunity elicited against COVID-19 by vaccination and natural infection is necessary to evaluate (10). The vaccine immunity might be diverse depending on the vaccine platform, vaccination interval, and the existence of natural infection. Thus, this study aimed to investigate the short-term immunogenicity, longevity, and cross-reactive neutralizing activity of Ad26.COV2.S against Omicron subvariants. We conducted a prospective longitudinal cohort study up to 6 months after homologous and heterologous booster vaccination in Ad26.COV2.S-primed individuals. We also compared the humoral and cellular immune responses between individuals with and without SARS-CoV-2 breakthrough infections during the study period.

## 2 Methods

### 2.1 Study design and procedures

**Figure 1:**
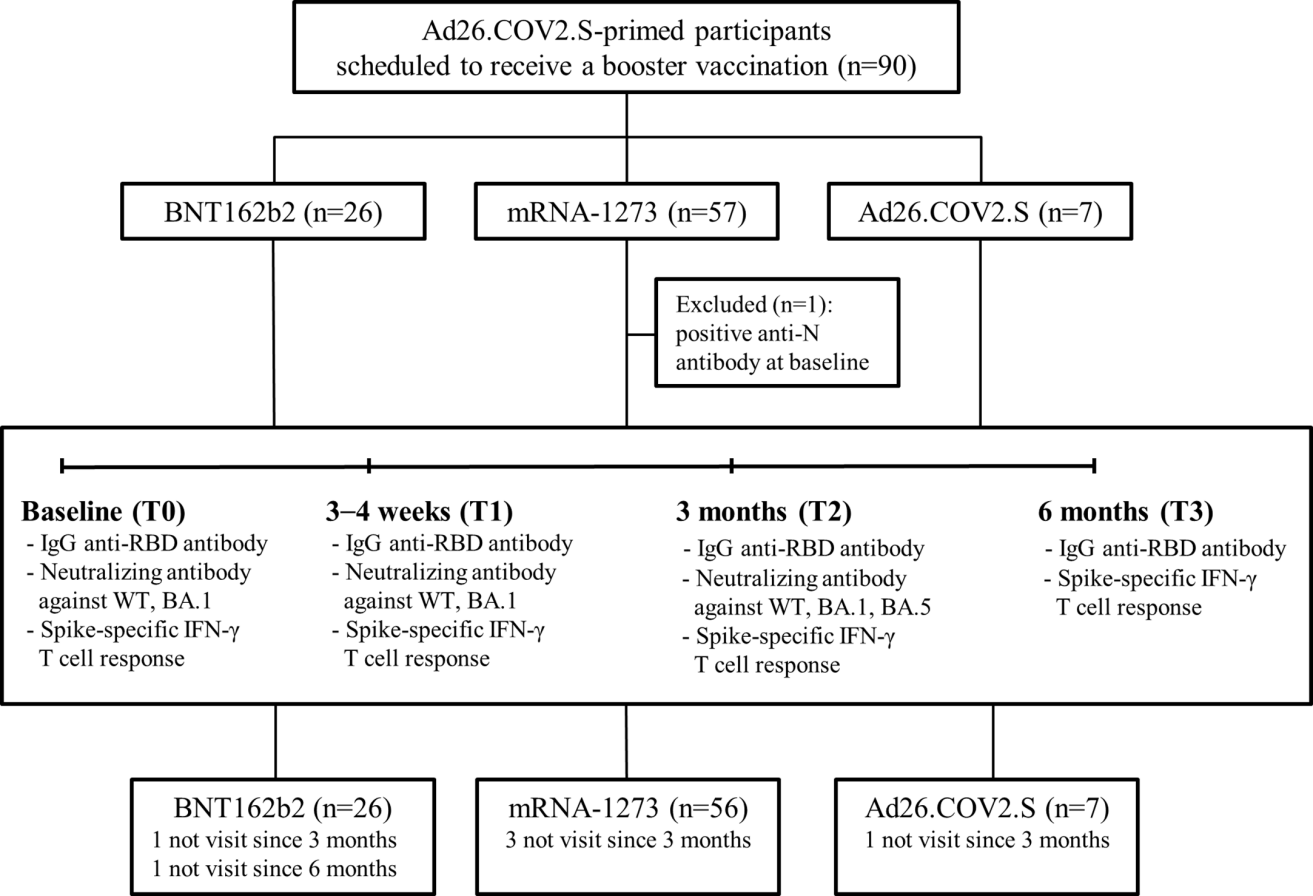
Study flowchart. Abbreviation: IgG, immunoglobulin G; RBD, receptor binding domain; WT, wild type.

This prospective multicenter cohort study was conducted from October 2021 to June 2022 in three tertiary university hospitals (Korea University Guro Hospital, Ajou University Hospital, and Hanllym University Hospital) in South Korea. Eligible participants were healthy adults who had received a primary dose of Ad26.COV2.S at least 5 months prior and were scheduled to receive a booster vaccine. The eligible booster vaccines were BNT162b2 (30 μg), mRNA-1273 (50 μg), and Ad26.COV2.S (5×10^10^ virus particles). As for the Ad26.COV2.S-boosted group, considering the small number of participants, we did not limit the interval for the inclusion criteria between the primary and booster doses. Those who had prior SARS-CoV-2 infection or immunocompromising conditions were excluded from this study. Individuals with a positive anti-nucleocapsid (N) protein antibody at baseline were also excluded. The flowchart of the study is shown in **Figure 1**. Blood samples were collected at baseline (T0, day of the booster dose), 3–4 weeks post-booster dose (T1), 3 months post-booster dose (T2), and 6 months post-booster dose (T3). The receptor binding domain (RBD) of SARS-CoV-2-specific immunoglobulin G (IgG) antibody and SARS-CoV-2-specific T-cell responses (against wild type [WT], Alpha [B.1.1.7], Beta [B.1.351], and Gamma [P.1]) were investigated at each time point. The neutralizing activities against WT and Omicron BA.1 were investigated at T0, T1, and T2. The neutralizing activities against Omicron BA.5 were investigated at T2. The neutralizing antibody (NAb) titers were measured in all participants from the BNT162b2- and Ad26.COV2.S-boosted groups and in 26 randomly selected participants from the mRNA-1273-boosted group (matched number to the BNT162b2-boosted group). The humoral and cellular immune responses of the participants from the three groups were compared at each time point. In addition, the peak post-booster immune status and immune responses derived from breakthrough infections were compared between SARS-CoV-2-uninfected and -infected individuals. We regarded the immune status at T1 as the peak post-booster immunity which was elicited by booster vaccination. To compare the peak post-booster immunity of the participants with respect to the breakthrough infections, ‘SARS-CoV-2-infected participants’ were defined as individuals with SARS-CoV-2 infection occurred between T1 and T2, while “SARS-CoV-2-uninfected participants’ were determined as those without breakthrough infection or with SARS-CoV-2 infection identified at T3.

This study was approved by the ethics committees of Korea University Guro Hospital (2021GR0099), Ajou University Hospital (AJIRB-BMR-SMP-21-528), and Hanllym University Hospital (202111026) and was conducted in accordance with the Declaration of Helsinki and Good Clinical Practice guidelines. Written informed consent was obtained from all participants.

### 2.2 Immunogenicity analysis

The Elecsys^®^ Anti-SARS-CoV-2 S immunoassay (Roche) was performed to measure IgG anti-RBD antibodies using a Cobas 8000 (Roche Diagnostics, Basel, Switzerland) in accordance with the manufacturer’s protocol. Anti-N antibodies were measured in each participant using a SARS-CoV-2 IgG assay (Abbott Laboratories, Chicago, IL, USA) to identify SARS-CoV-2 infection.

For the NAbs analysis, a plaque reduction neutralization test was performed using WT SARS-CoV-2 (βCoV/Korea/KCDC03/2020 NCCP No. 43326), Omicron BA.1 subvariant (GRA: B.1.1.529 NCCP No. 43408), and Omicron BA.5 subvariant (GRA: BA.5 NCCP No. 43426). Briefly, a mixture of serum dilution/virus (40 PFU/well) was incubated at 37°C for 2 h, added to the plate seeded with Vero E6 cells, incubated at 37°C for 1 h, and then added with 0.5% agarose (Lonza, Basel, Switzerland). After 2–3 days of incubation, the cells were fixed with 4% paraformaldehyde and stained to visualize plaques. A reduction in plaque count of 50% was then calculated for the median neutralizing titer (ND_50_) using the Spearman–Karber formula, and ND_50_ ≥ 1:20 was considered positive.

SARS-CoV-2-specific T-cell responses were evaluated using Covi-FERON FIA (SD Biosensor, Suwon, Korea), a fluorescence immunoassay (FIA) for detecting interferon-γ (IFN-γ) secreted by T cells in response to SARS-CoV-2-specific proteins, in accordance with the manufacturer’s instructions. Whole blood was collected in heparinized tubes, which included Nil tubes (negative control), original S protein antigen tubes, variant S protein tubes, N protein tubes, and mitogen tubes (positive control). The original S protein antigen tube included antigens derived from the WT and Alpha variants (lineage B.1.1.7, 20I/501Y. V1) of SARS-CoV-2. The variant S protein tube contained antigens derived from the Beta (lineage B.1.351, 20H/501. V2) and Gamma (lineage P.1, 20 J/501Y. V3) variants of SARS-CoV-2. Blood samples were incubated at 37°C for 16–24 h and then centrifuged for 15 min at a relative centrifugal force of 2200–2300 gravity. After centrifugation, plasma was collected, and the amount of IFN-γ was measured using FIA. The cut-off value of IFN-γ was 0.25 IU/mL.

### 2.3 Statistical analysis

Humoral and cellular immune responses were compared among the three groups at each time point. The chi-square test or Fisher’s exact test was used to compare categorical variables, and the Kruskal–Wallis test was used for continuous variables to compare the differences between the three groups. To analyze the longevity of humoral and cellular immune responses in SARS-CoV-2-naive participants, we excluded participants who had breakthrough infections at each time point. The geometric mean titer (GMT) with 95% confidence interval (CI) was calculated after logarithmic transformation of the antibody titers. The Wilcoxon signed-rank test was used to compare paired data, and the Mann–Whitney U test was used to compare unpaired data. Statistical analysis was performed using the Statistical Package for the Social Sciences version 20 (SPSS Inc., Chicago, IL, USA) or GraphPad Prism software (version 9.0; GraphPad Software, Inc., San Diego, CA, USA). Statistical significance was set at *P* < 0.05.

## 3 Results

### 3.1 Study participants

A total of 90 participants were recruited in this study: 26 boosted with BNT162b2, 57 with mRNA-1273, and 7 with Ad26.COV2.S. One participant from the mRNA-1273-boosted group had a positive result for N protein antibody at baseline and thus was excluded (**Figure 1**). The characteristics of the participants are listed in **Table 1**. The majority of the participants were male (90%) under the age of 40 years (median age, 34 years; interquartile range [IQR], 32–37). The median intervals between the primary and booster doses of the participants from the BNT162b2-, mRNA-1273-, and Ad26.COV2.S-boosted groups were 27 weeks (IQR, 25–28), 25 weeks (IQR, 24–27), and 23 weeks (IQR, 15–24), respectively. During the study period, the rate of laboratory-confirmed breakthrough infections did not differ among the three groups (42.9–62.5%; **Table 1**). All breakthrough infections occurred 3–6 months after booster vaccination during follow-up. Five participants, including one from the BNT162b2-, three from the mRNA-1273-, and one from the Ad26.COV2.S-boosted group, were lost to follow-up 3 months after vaccination. One participant from the BNT162b2-boosted group was lost to follow-up at 6 months post-vaccination (**Figure 1**).

**Table 1:** Characteristics of study participants.

	BNT162b2 (N = 26)	mRNA-1273 (N = 56)	Ad26.COV2.S (N = 7)	*P*-value
Male, No. (%)	26 (100)	50 (89.3)	4 (57.1)	NA
Median age (IQR), years	36 (34–36)	34 (32–37)	35 (33–41)	0.110
Median interval between priming and booster dose (IQR), weeks	27 (25–28)	25 (24–27)	23 (15–24)	<0.001
Breakthrough infection, No. (%)	14 (53.8)	35 (62.5)	3 (42.9)	0.521
Male, No. (%)	14 (53.8)	30 (53.6)	3 (42.9)	NA
Median age (IQR), years	36 (34–38)^a^	33 (32–36)^b^	34 (33–34)^ab^	0.016
Timing of breakthrough infection				0.857
Confirmed at 3–4 weeks, No. (%)	0 (0)	0 (0)	0 (0)	
Confirmed at 3 months, No. (%)^*^	5 (19.2)	13 (23.2)	1 (14.3)	
Confirmed at 6 months, No. (%)^†^	9 (34.6)	22 (39.3)	2 (28.6)	

^*^Five participants (one from the BNT162b2-boosted group, three from the mRNA-1273-boosted group, and one from the Ad26.COV2.S-boosted group) were lost to follow-up.

^†^Six participants (two from the BNT162b2-boosted group, three from the mRNA-1273-boosted group, and one from the Ad26.COV2.S-boosted group) were lost to follow-up.

The values with different superscript letters in a column are significantly different (P < 0.05)

No, number; NA, not applicable; IQR, interquartile range.

### 3.2 Humoral immune response

**Figure 2:**
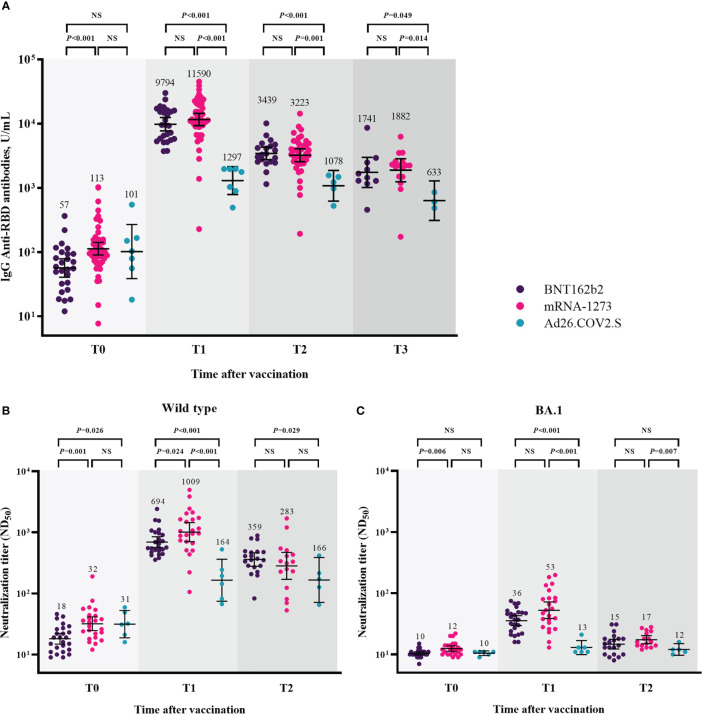
Humoral immune responses after booster vaccination. GMTs of IgG anti-RBD antibodies **(A)**, ND_50_ against wild type virus **(B)**, and ND_50_ against Omicron BA.1 **(C)**. Blood samples were collected at baseline (day of booster dose, T0), 3–4 weeks post-booster dose (T1), 3 months post-booster dose (T2), and 6 months post-booster dose (T3). The black bar represents GMT with 95% confidence intervals. Abbreviation: NS, not significant; IgG, immunoglobulin G; GMT, geometric mean titer; RBD, receptor binding domain; ND_50_, 50% neutralization dose.

Humoral immune responses after booster vaccination are shown in **Figure 2** and **Supplementary Figure 1**. Humoral immune responses were compared among the three groups, excluding participants with breakthrough infections at each time point. At T0, the GMTs of IgG anti-RBD antibodies were lower in the participants from the BNT162b2-boosted group than in those from the mRNA-1273-boosted group (57 [95% CI, 41–79] vs. 113 [95% CI, 90–142], *P* < 0.001). The GMT of IgG anti-RBD antibodies was significantly increased at T1, and participants in all three groups maintained higher titers of IgG anti-RBD antibodies at T3 compared to T0 (**Supplementary Figure 1A**). However, the GMTs of IgG anti-RBD antibodies after booster vaccination were significantly lower in the participants from the Ad26.COV2.S-boosted group than in those from the mRNA vaccine-boosted groups at each time point (**Figure 2A**). No significant difference in the GMTs of IgG anti-RBD antibodies after booster vaccination was found between the participants from the BNT162b2- and mRNA-1273-boosted groups at each time point. The GMTs of IgG anti-RBD antibodies and respective *P*-value among three groups were shown in the **Supplementary Table 1**.

The neutralizing activity against WT after booster vaccination is shown in **Figure 2B**. The GMTs of NAb against WT were significantly lower in the participants from the BNT162b2-boosted group than in those from the mRNA-1273- and Ad26.COV2.S-boosted groups at T0 (BNT162b2 group vs. mRNA-1273 group vs. Ad26.COV2.S group, 18 vs. 32 vs. 31, *P* = 0.003). At T1, the GMTs of NAb were 694, 1009, and 164 in the participants from the BNT162b2-, mRNA-1273-, and Ad26.COV2.S-boosted groups, respectively (*P* < 0.001). At T2, the GMTs of NAb did not significantly differ between the participants from the mRNA vaccine-boosted groups (359 vs. 283, *P* = 0.408). The participants from the BNT162b2-boosted group had significantly higher NAb titers than those from the Ad26.COV2.S-boosted group (359 vs. 166, *P* = 0.029). The GMTs of NAb against BA.1 were negligibly low at T0 in all participants from the three groups (**Figure 2C**). Compared with the baseline (T0) levels, the neutralizing activities against BA.1 significantly increased in the participants from the BNT162b2- and mRNA-1273-boosted groups (*P* < 0.001) but not in those from the Ad26.COV2.S-boosted group at T1 and T2 (**Supplementary Figure 1C**). The GMTs of NAb against BA.1 were significantly lower in the participants from the Ad26.COV2.S-boosted group than in those from the mRNA-1273-boosted group at each time points after booster vaccination (**Figure 2C**). The GMTs of NAb against WT/BA.1 and respective *P*-value among three groups were shown in the **Supplementary Table 1**.

**Figure 3:**
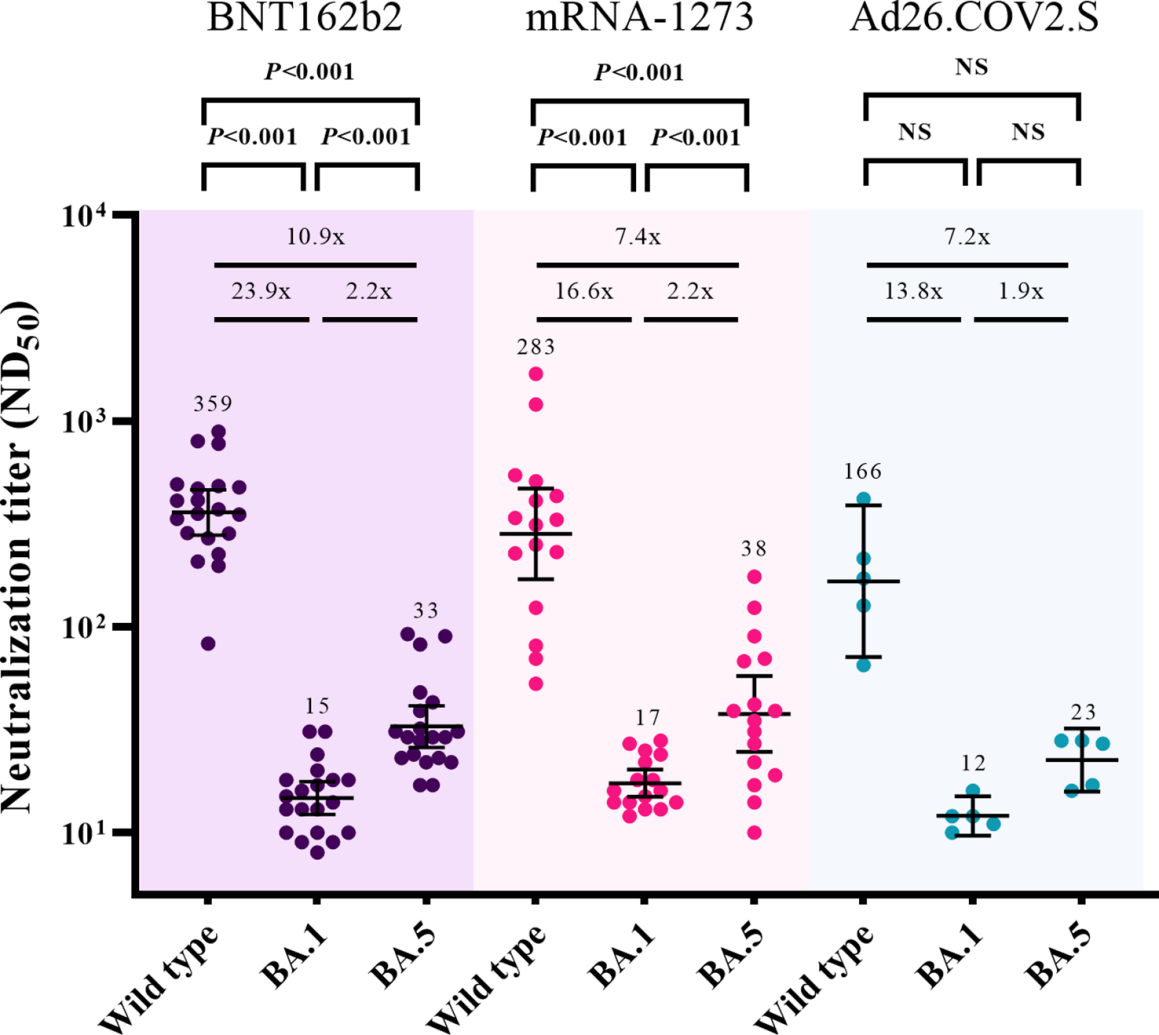
Comparisons of neutralizing activities against wild type, Omicron BA.1, and Omicron BA.5 viruses at 3 months after booster vaccination. The black bar represents GMT with 95% confidence intervals. NS, not significant; ND_50_, 50% neutralization dose; GMT, geometric mean titer.

The cross-neutralizing activities against Omicron subvariants (BA.1 and BA.5) were assessed 3 months after booster vaccination (T2) (**Figure 3**). After excluding participants with breakthrough infections, 20, 16, and 5 participants from the BNT162b2-, mRNA-1273-, and Ad26.COV2.S-boosted groups, respectively, were included in the analysis. Compared with the neutralizing activities against WT, those against BA.1 were lower by 23.9-fold (359 vs. 15, *P* < 0.001), 16.6-fold (283 vs. 17, *P* < 0.001), and 13.8-fold (166 vs. 12, *P* = 0.063) in the participants from the BNT162b2-, mRNA-1273-, and Ad26.COV2.S-boosted groups, respectively. The neutralizing activities against BA.5 versus WT were lower by 10.9-fold (359 vs. 33, *P* < 0.001), 7.4-fold (283 vs. 38, *P* < 0.001), and 7.2-fold (166 vs. 23, *P* = 0.063) in the participants from the BNT162b2-, mRNA-1273-, and Ad26.COV2.S-boosted groups, respectively. The GMTs of NAb against BA.5 were higher than those against BA.1 by 2.2-fold (33 vs. 15, *P* < 0.001), 2.2-fold (38 vs. 17, *P* < 0.001), and 1.9-fold (23 vs. 12, *P* = 0.063) in the participants from the BNT162b2-, mRNA-1273-, and Ad26.COV2.S-boosted groups, respectively.

### 3.3 SARS-CoV-2-specific IFN-γ T cell response

**Figure 4:**
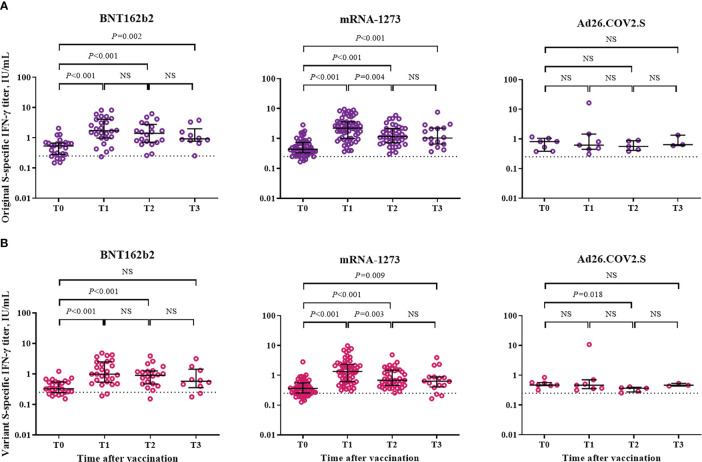
Cellular immune response after booster vaccination. **(A)** SARS-CoV-2 original spike protein-specific interferon-γ release assay. **(B)** SARS-CoV-2 variant spike protein-specific interferon-γ release assay. Blood samples were collected at baseline (day of booster dose, T0), 3–4 weeks post-booster dose (T1), 3 months post-booster dose (T2), and 6 months post-booster dose (T3). The black bar represents median with interquartile range. NS, not significant; S, spike; IFN- γ, interferon gamma.

The cellular immune response was assessed based on IFN-γ response against the S protein of SARS-CoV-2 by using a Covi-FERON ELISA kit (**Figure 4**). Positivity for IFN-γ response against the original S antigen (WT and Alpha) was observed in 81% (21/26), 93% (53/57), and 100% (7/7) of the participants from the BNT162b2-, mRNA-1273-, and Ad26.COV2.S-boosted groups at T0, respectively. As for the variant S antigens (Beta and Gamma), positivity for IFN-γ response was observed in 77% (20/26), 81% (46/57), and 100% (7/7) of the participants from the BNT162b2-, mRNA-1273-, and Ad26.COV2.S-boosted groups at T0, respectively. Compared with the baseline (T0) levels, the IFN-γ response against the original S antigen significantly increased at T1 and was sustained at T3 in the participants from the BNT162b2- and mRNA-1273-boosted groups but not in those from the Ad26.COV2.S-boosted group (**Figure 4A**). As for the IFN-γ response against variant S antigens, the significant increase in IFN-γ response was sustained at T2 and T3 in the participants from the BNT162b2- and mRNA-1273-boosted groups, respectively (**Figure 4B**). IFN-γ responses against original and variant S antigens did not differ between mRNA vaccine-boosted groups. However, mRNA-1273-boosted group showed significantly higher IFN-γ responses against original and variant S antigens compared to Ad26.COV2.S-boosted group at T1 and T2 (**Supplementary Table 1**).

### 3.4 Comparison of immune responses between SARS-CoV-2-infected and -uninfected participants

**Figure 5:**
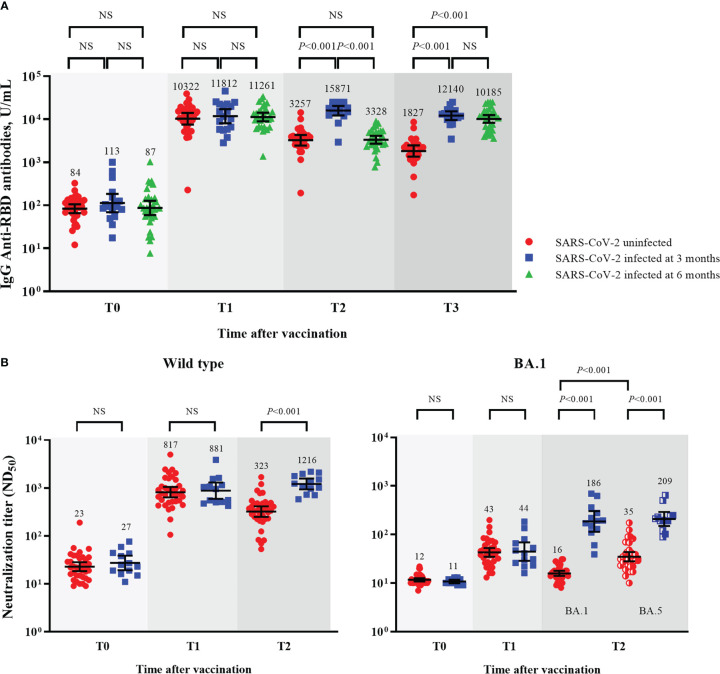
Comparisons of humoral immunity between SARS-CoV-2-uninfected and -infected participants among mRNA vaccine-boosted group. GMTs of IgG anti-RBD antibodies **(A)**, and ND_50_ against wild type and Omicron BA.1 viruses **(B)**. Blood samples were collected at baseline (day of booster dose, T0), 3–4 weeks post-booster dose (T1), 3 months post-booster dose (T2), and 6 months post-booster dose (T3). The black bar represents GMT with 95% confidence intervals. NS, not significant; IgG, immunoglobulin G; GMT, geometric mean titer; RBD, receptor binding domain; ND_50_, 50% neutralization dose.

At 6 months follow-up, SARS-CoV-2 breakthrough infections occurred in 53.8% (14/26), 62.5% (35/56), and 42.9% (3/7) of the participants from the BNT162b2-, mRNA-1273-, and Ad26.COV2.S-boosted groups, respectively. All cases of breakthrough infections were mild in severity and did not require hospitalization. Of the 52 cases of SARS-CoV-2 breakthrough infections, 19 and 33 cases were identified at T2 and T3, respectively (**Table 1**). The Ad26.COV2.S-boosted group had considerably fewer participants to compare the difference between SARS-CoV-2-infected and -uninfected groups. Thus, the comparison was conducted only among the participants from the mRNA vaccine-boosted groups. No laboratory-confirmed cases of COVID-19 were recorded at T1. The IgG anti-RBD antibody and IFN-γ responses against the original and variant S proteins at T1 were compared between 64 SARS-CoV-2-uninfected participants (33 without and 31 with SARS-CoV-2 breakthrough infections at T3) and 18 SARS-CoV-2-infected participants (SARS-CoV-2 breakthrough infections identified at T2). The neutralizing activities against WT and Omicron subvariants were compared between 36 SARS-CoV-2-uninfected participants (16 without and 20 with SARS-CoV-2 breakthrough infections identified at T3) and 13 SARS-CoV-2-infected participants. The neutralization assay was conducted only in selected participants from the mRNA-1273-boosted group age-matched with those from the BNT162b2-boosted group. Hence, a discrepancy in the number of participants was noted in some analyses. There was no difference in peak post-booster humoral immunity (IgG anti-RBD antibodies [10766 vs. 11812, *P* = 0.702], NAb against WT [817 vs. 881, *P* = 0.885], and BA.1 [43 vs. 44, *P* = 0.969]) and cellular immunity (original S antigen [2.20 vs. 1.77, *P* = 0.810] and variant S antigens [1.28 vs. 1.27, *P* = 0.760]) between the SARS-CoV-2-infected and -uninfected participants (**Table 2**; **Figure 5** and **Supplementary Figure 2**).

**Table 2:** Comparisons of peak post-booster humoral and cellular immunities at 3–4 weeks after booster vaccination between SARS-CoV-2-uninfected and -infected participants^*^.

	SARS-CoV-2 uninfected	SARS-CoV-2 infected	*P*-value
IgG anti-RBD antibodies GMT (95% CIs), U/mL^†^	10766 (8895–13031)	11812 (8087–17251)	0.702
NAb against WT GMT (95% CIs)^‡^	817 (637–1048)	881 (596–1302)	0.885
NAb against BA.1 GMT (95% CIs)^‡^	43 (35–53)	44 (29–69)	0.969
IGRA against original spike median (IQR), IU/mL^†§^	2.20 (1.02–3.51)	1.77 (0.93–5.18)	0.810
IGRA against variant spike median (IQR), IU/mL^†¶^	1.28 (0.64–2.36)	1.27 (0.50–3.22)	0.760

^*^The Ad26.COV2.S-boosted group was excluded from analysis.

^†^The numbers of SARS-CoV-2-uninfected and-infected participants were 64 and 18, respectively.

^‡^The numbers of SARS-CoV-2-uninfected and-infected participants were 36 and 13, respectively. The reason for the decreased number of participants was that the neutralizing antibody assay was conducted in randomly selected participants from the mRNA-1273-boosted group.

^§^Original spike protein derived from wild type and Alpha variants of SARS-CoV-2.

^¶^Variant spike protein derived from the Beta and Gamma variants of SARS-CoV-2.

IgG, immunoglobulin G; RBD, receptor binding domain; GMT, geometric mean titer; WT, wild type; CI, confidence interval; NAb, neutralizing antibody; IGRA, interferon gamma release assay; IQR, interquartile range.

After breakthrough infections, the SARS-CoV-2-infected participants had higher GMTs of IgG anti-RBD antibodies and NAb than the SARS-CoV-2-uninfected participants (**Figures 5A, B**). The GMTs of NAb against BA.5 also increased after the breakthrough infections. The neutralizing activities against BA.5 were higher than those against BA.1 in the SARS-CoV-2-uninfected participants (35 vs. 16, *P* < 0.001) but not in the SARS-CoV-2-infected participants (186 vs. 204, *P* = 0.724; **Figure 5B**).

## 4 Discussion

The key findings of this study are as follows: (1) a heterologous booster dose of mRNA vaccine in the Ad26.COV2.S-primed population induced good and sustained humoral and cellular immune responses for up to 6 months; (2) despite booster vaccination, the neutralizing activities against Omicron subvariants were negligible in the Ad26.COV2.S-primed population; (3) breakthrough infections after booster vaccination elicited acceptable NAb against Omicron subvariants; and (4) no difference in baseline humoral and cellular immunities was noted between individuals with and without breakthrough infections.

The Ad26.COV2.S vaccine showed acceptable efficacy and durable humoral/cellular immune responses in clinical trials (6). In the present study, homologous and heterologous booster vaccinations in the Ad26.COV2.S-primed participants were immunogenic. However, the quantities of IgG anti-RBD antibodies and NAbs were significantly higher in the heterologous booster groups than in the homologous booster group. In addition, T-cell immunity was enhanced for up to 6 months after heterologous booster vaccination but not after homologous booster vaccination. These results are consistent with those of previous studies (11–13). Repeated exposure to the adenovirus vector in homologous booster vaccination might result in diminished immune responses. In this aspect, heterologous Ad26.COV2.S-mRNA vaccine booster vaccination would be more immunogenic than homologous booster vaccination in the Ad26.COV2.S-primed population. Notably, the participants from the mRNA-1273-boosted group showed higher neutralizing activities (GMTs) against WT than those from the BNT162b2-boosted group at 3–4 weeks after booster vaccination (T1) (1009 vs. 694, *P* = 0.024). This result can be ascribed to the higher pre-booster titers of the participants from the mRNA-1273-boosted group than those of the participants from the BNT162b2-boosted group (32 vs. 18, *P* = 0.001). However, the difference between these participants became negligible 3 months after booster vaccination (T2).

As diverse SARS-CoV-2 variants of concern (VOCs) emerge and spread, cross-reactive neutralizing activities against VOCs are important to predict vaccine efficacy (14). Although the immune correlation of protection against SARS-CoV-2 infection is not well established, NAb titers may play a key role in protection against SARS-CoV-2 infection (15). A predictive model showed that a normalized neutralization titer of 68 is associated with 50% protection against symptomatic SARS-CoV-2 infection (16). Another study on healthcare workers reported that NAb titers of 64–128 provide 94% protection against COVID-19 (17). In the present study, the cross-reactive neutralizing activities against Omicron subvariants were negligibly low at baseline (ND_50_, 10–23). After heterologous booster vaccination, the neutralizing activities against Omicron BA.1 slightly increased at 3–4 weeks (T1), with titers of 58 and 36 in the mRNA-1273- and BNT162b2-boosted groups, respectively. In addition, NAb titers decayed to undetectable levels 3 months after booster vaccination (T2). Moreover, the NAb response against Omicron BA.1 was not enhanced after homologous booster vaccination with Ad26.COV2.S. This result suggests that the neutralizing activities against Omicron BA.1 elicited by a single dose of the booster vaccine could not provide sufficient protection against Omicron subvariants in the Ad26.COV2.S-primed population. Additional doses of booster or bivalent booster vaccinations should be considered for this population.

Omicron BA.5 is one of three lineages (BA.2.12.1, BA.4, and BA.5) derived from BA.2. Different from BA.2, Omicron BA.5 has additional mutations of L452R, F486V, and R493Q in the spike RBD (18). Recent studies have found low cross-reactive neutralization between Omicron BA.1 and BA.5 (19–21). Furthermore, the hybrid immunity elicited by Omicron BA.1 breakthrough infection can be evaded by Omicron BA.4/BA.5 because of the spike RBD mutations of L452Q, L452R, and F486V (20, 22, 23). In the present study, the neutralizing activities against BA.5 were low but still higher than those against BA.1 in the mRNA vaccine-boosted participants without breakthrough infections. In addition, after breakthrough infections, the neutralizing activities against BA.1 and BA.5 were enhanced in the heterologously boosted participants. Most cases of breakthrough infections in the present study developed in early 2022 when Omicron BA.1 was the predominant strain, explaining the remarkable cross-reactive neutralizing activities between Omicron subvariants BA.1 and BA.5, contrary to previous reports (19–24). Immune responses after vaccination and natural infection may vary depending on age, sex, race, vaccine type/dose, and SARS-CoV-2 strain (25, 26).

With respect to the breakthrough infection, there was no significant difference in the peak post-booster humoral and cellular immunity between SARS-CoV-2-infected and -uninfected participants. The frequency, intensity, and duration of viral exposure and the predominant strain of SARS-CoV-2 variants at the time of exposure may be important factors in determining the occurrence of breakthrough infections. Hybrid immunity elicited by natural infection and booster vaccination showed better neutralizing activity against WT and Omicron subvariants, but its longevity warrants further investigation.

This study has some limitations. First, most study participants were young and male. According to the policy of the Korean government, the Ad26.COV2.S vaccine was first administered in South Korea to military reservists aged 30–60 years, explaining why most of the participants were young men. As immune responses after vaccination can be diverse according to sex and age, the data in this study should be cautiously generalized. The immune responses in the elderly who are vulnerable to severe COVID-19 might be different from those in our study participants. Second, the cellular immune responses against SARS-CoV-2 Omicron subvariants were not evaluated. The cellular immune responses against Omicron differ from those against other variants. Third, the neutralization assay at 6 months post-booster vaccination was not conducted. Due to limitations in time and labor, neutralizing antibody tests could be only performed at limited points in time. Data on NAb titers against Omicron BA.1/BA.5 in SARS-CoV-2-infected and -uninfected participants at 6 months post-booster vaccination would be useful in establishing vaccination strategy. On the other hand, the strength of this study is that we conducted a serial estimation of humoral and cellular immune responses after booster vaccination in the Ad26.COV2.S-primed population for up to 6 months. In addition, a neutralization assay was performed against the Omicron subvariants.

In conclusion, heterologous booster vaccination is recommended for the Ad26.COV2.S-primed population. However, a single dose of heterologous mRNA vaccine booster was not sufficient to provide protection against Omicron subvariants in SARS-CoV-2 infection-naïve and Ad26.COV2.S-primed individuals. Therefore, additional booster vaccinations may be required.

## Data availability statement

The original contributions presented in the study are included in the article/**Supplementary Material**. Further inquiries can be directed to the corresponding authors.

## Ethics statement

The studies involving human participants were reviewed and approved by The ethics committees of Korea University Guro Hospital The ethics committees of Ajou University Hospital The ethics committees of Hanllym University Hospital. The patients/participants provided their written informed consent to participate in this study.

## Author contributions

Conceptualization: HH, M-SP, and JYS. Data curation: HH, JH, YS, M-SP, and JYS. Formal analysis: HH, A-YJ, M-SP, and JYS. Funding acquisition: JYS. Investigation: HH, A-YJ, JH, YS, EN, JY, HS, JY, HP, JHS, JK, M-SP, and JYS. Methodology: HH, A-YJ, JN, M-SP, and JYS. Project administration: M-SP and JYS. Validation: HH, HC, JK, M-SP, and JYS. Visualization: HH and JYS. Writing - original draft: HH and JYS. Writing - review and editing: All authors. All authors contributed to the article and approved the submitted version.
