# Supplementary figures and images for: Humoral and cellular immunogenicity of homologous and heterologous booster vaccination in Ad26.COV2.S-primed individuals: Comparison by breakthrough infection
Source: Front Immunol. 2023 Mar 7;14:1131229. doi: 10.3389/fimmu.2023.1131229 (PMC10027912; doi:10.3389/fimmu.2023.1131229)

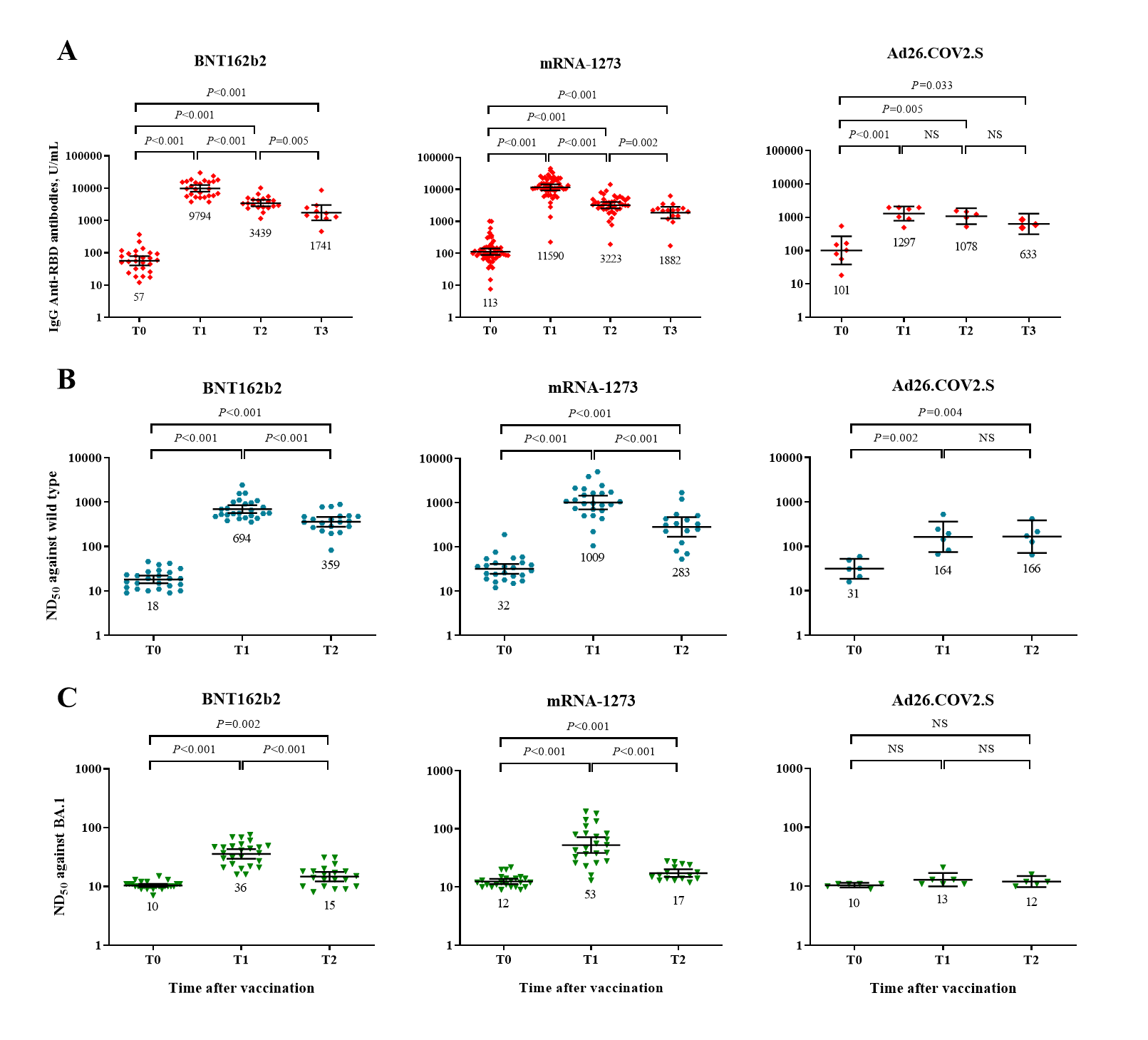

Supplement: Supplementary file 1 [file Image_1.tif]

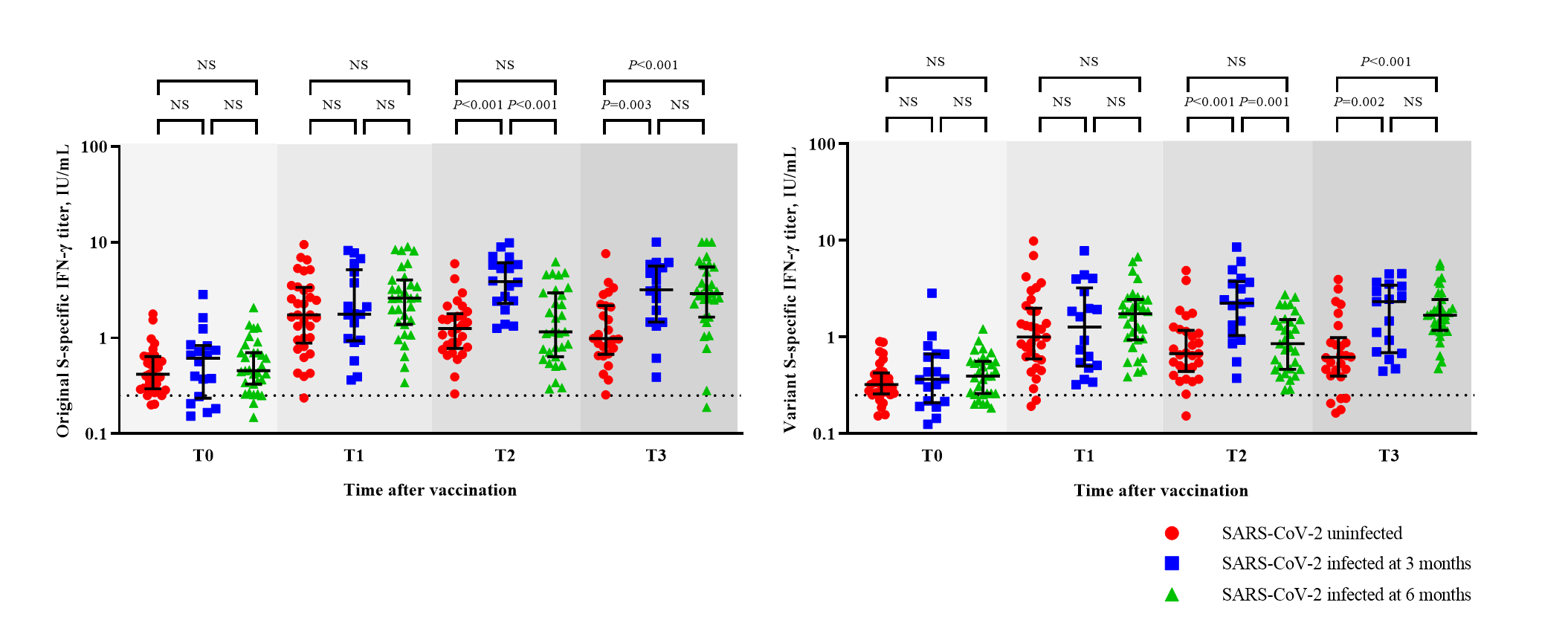

Supplement: Supplementary file 2 [file Image_2.tif]
