# Supplementary material for: Humoral and cellular immunogenicity of homologous and heterologous booster vaccination in Ad26.COV2.S-primed individuals: Comparison by breakthrough infection
Source: Front Immunol. 2023 Mar 7;14:1131229. doi: 10.3389/fimmu.2023.1131229 (PMC10027912; doi:10.3389/fimmu.2023.1131229)
Supplement: Supplementary file 3 [file Table_1.docx]

Supplementary Material

Humoral and cellular immunogenicity of homologous and heterologous booster vaccination in Ad26.COV2.S-primed individuals: Comparison by breakthrough infection

**Hakjun Hyun, A-Yeung Jang, Heedo Park, Jung Yeon Heo, Yu Bin Seo, Eliel Nham, Jin Gu Yoon, Hye Seong, Ji Yun Noh, Hee Jin Cheong, Woo Joo Kim, Soo-Young Yoon, Jong Hyeon Seok, Jineui Kim, Man-Seong Park*, Joon Young Song***

**Correspondence:** Man-Seong Park: [ms0392@korea.ac.kr](mailto:ms0392@korea.ac.kri) and Joon Young Song: [infection@korea.ac.kr](mailto:infection@korea.ac.kr)

**Supplementary Table 1.** Intergroup comparison of humoral and cellular immune responses after booster vaccination of COVID-19-naïve participants

|  | **Booster vaccine** | | |  | ***P*-value** | | |
| --- | --- | --- | --- | --- | --- | --- | --- |
| **Time after**  **vaccination^*^** | **BNT162b2** | **mRNA-1273** | **Ad26.COV2.S** |  | **BNT162b2 vs.**  **mRNA-1273** | **mRNA-1273**  **vs.**  **Ad26.COV2.S** | **Ad26.COV2.S**  **vs.**  **BNT162b2** |
| IgG anti-RBD antibody titers, GMT (95% CIs), U/mL | | | | | | | |
| T0 | 57 (41–79) | 113 (90–142) | 101 (39–269) |  | <0.001 | 0.922 | 0.120 |
| T1 | 9794 (7721–12424) | 11590 (9287–14465) | 1297 (789–2132) |  | 0.127 | <0.001 | <0.001 |
| T2 | 3439 (2748–4304) | 3223 (2552–4070) | 1078 (622–1867) |  | 0.944 | 0.001 | <0.001 |
| T3 | 1741 (1014–2990) | 1882 (1245–2846) | 633 (312–1284) |  | 0.363 | 0.014 | 0.049 |
| 50% neutralization dose for WT, GMT (95% CIs) | | | | | | | |
| T0 | 18 (14–23) | 32 (25–41) | 31 (19–53) |  | 0.001 | 0.970 | 0.026 |
| T1 | 694 (567–849) | 1009 (705–1444) | 164 (74–363) |  | 0.024 | <0.001 | <0.001 |
| T2 | 359 (278–463) | 283 (171–470) | 166 (71–389) |  | 0.408 | 0.208 | 0.029 |
| 50% neutralization dose for BA.1, GMT (95% CIs) | | | | | | | |
| T0 | 10 (10–11) | 12 (11–14) | 10 (10–11) |  | 0.006 | 0.094 | 0.671 |
| T1 | 36 (29–43) | 53 (39–72) | 13 (10–17) |  | 0.051 | <0.001 | <0.001 |
| T2 | 15 (12–18) | 17 (15–20) | 12 (10–15) |  | 0.201 | 0.007 | 0.292 |
| Original spike-specific IFN-γ titer, median (IQR), IU/mL^†^ | | | | | | | |
| T0 | 0.54 (0.28–0.67) | 0.43 (0.33–0.74) | 0.82 (0.39–1.05) |  | 0.757 | 0.142 | 0.169 |
| T1 | 1.71 (0.98–4.10) | 2.21 (1.00–3.59) | 0.62 (0.45–1.47) |  | 0.453 | 0.021 | 0.120 |
| T2 | 1.40 (0.71–2.80) | 1.15 (0.72–2.16) | 0.56 (0.41–0.88) |  | 0.861 | 0.026 | 0.069 |
| T3 | 0.93 (0.77–2.00) | 1.04 (0.66–2.23) | 0.64 (0.59–1.35) |  | 0.897 | 0.359 | 0.371 |
| Variant spike-specific IFN-γ titer, median (IQR), IU/mL^‡^ | | | | | | | |
| T0 | 0.33 (0.25–0.54) | 0.34 (0.26–0.55) | 0.47 (0.45–0.57) |  | 0.920 | 0.045 | 0.155 |
| T1 | 0.99 (0.54–2.47) | 1.37 (0.78–2.45) | 0.46 (0.36–0.70) |  | 0.294 | 0.023 | 0.082 |
| T2 | 0.90 (0.47–1.26) | 0.66 (0.46–1.20) | 0.37 (0.28–0.39) |  | 0.452 | 0.005 | 0.010 |
| T3 | 0.57 (0.36–1.43) | 0.63 (0.41–0.84) | 0.46 (0.44–0.54) |  | 0.853 | 0.217 | 0.692 |

^*^T0, baseline; T1, at 3–4 weeks post-booster vaccination; T2, at 3 months post-booster vaccination; T3, at 6 months post-booster vaccination.

^†^Original spike derived from wild type and Alpha variants of SARS-CoV-2.

^‡^Variant spike derived from Beta and Gamma variants of SARS-CoV-2.

*P*-value resulting from Mann–Whitney U test.

Abbreviations: IgG, immunoglobulin G; RBD, receptor binding domain; GMT, geometric mean titer; CI, confidence interval; WT, wild type; IFN-γ, interferon gamma; IQR, interquartile range.

**Supplementary Figure 1.** Humoral immune response after booster vaccination. GMTs of IgG anti-RBD antibodies (A), ND_50_ against wild-type virus (B), and ND_50_ against Omicron BA.1 (C). Blood samples were collected at baseline (day of booster dose, T0), 3–4 weeks post-booster dose (T1), 3 months post-booster dose (T2), and 6 months post-booster dose (T3). The black bar represents GMT with 95% confidence intervals. Abbreviations: NS, not significant; IgG, immunoglobulin G; GMT, geometric mean titer; RBD, receptor binding domain; ND_50_, 50% neutralization dose; WT, wild type.

**Supplementary Figure 2.** Cellular immune response after booster vaccination by mRNA vaccine. (*A*) SARS-CoV-2 original spike protein-specific interferon-γ release assay. (*B*) SARS-CoV-2 variant spike protein-specific interferon-γ release assay. Blood samples were collected at baseline (day of booster dose, T0), 3–4 weeks post-booster dose (T1), 3 months post-booster dose (T2), and 6 months post-booster dose (T3). The black bar represents median with interquartile range. Abbreviations: NS, not significant; S, Spike; IFN-γ, interferon gamma.
